# Community phytosanitation to manage cassava brown streak disease

**DOI:** 10.1016/j.virusres.2017.04.020

**Published:** 2017-09-15

**Authors:** James Legg, Mathias Ndalahwa, Juma Yabeja, Innocent Ndyetabula, Hein Bouwmeester, Rudolph Shirima, Kiddo Mtunda

**Affiliations:** a International Institute of Tropical Agriculture, PO Box 34441, Dar es Salaam, Tanzania; b Maruku Agricultural Research Institute, PO Box 127, Bukoba, Tanzania; c Geospace, Roseboomlaan 38, 6717 ZB Ede, Netherlands; d Sugarcane Agricultural Research Institute, PO Box 30031, Kibaha, Tanzania

**Keywords:** Cassava brown streak virus, Collective action, Epidemiology, GIS, Tanzania, Whitefly

## Abstract

Cassava viruses are the major biotic constraint to cassava production in Africa. Community-wide action to manage them has not been attempted since a successful cassava mosaic disease control programme in the 1930s/40 s in Uganda. A pilot initiative to investigate the effectiveness of community phytosanitation for managing cassava brown streak disease (CBSD) was implemented from 2013 to 2016 in two communities in coastal (Mkuranga) and north-western (Chato) Tanzania. CBSD incidence in local varieties at the outset was >90%, which was typical of severely affected regions of Tanzania. Following sensitization and monitoring by locally-recruited taskforces, there was effective community-wide compliance with the initial requirement to replace local CBSD-infected material with newly-introduced disease-free planting material of improved varieties. The transition was also supported by the free provision of additional seed sources, including maize, sweet potato, beans and cowpeas. Progress of the initiative was followed in randomly-selected monitoring fields in each of the two locations. Community phytosanitation in both target areas produced an area-wide reduction in CBSD incidence, which was sustained over the duration of the programme. In Chato, maximum CBSD incidence was 39.1% in the third season, in comparison with an incidence of >60% after a single season in a control community where disease-free planting material was introduced in the absence of community phytosanitation. Kriging and geospatial analysis demonstrated that inoculum pressure, which was a function of vector abundance and the number of CBSD-infected plants surrounding monitored fields, was a strong determinant of the pattern of CBSD development in monitored fields. In the first year, farmers achieved yield increases with the new varieties relative to the local variety baseline of 94% in Chato (north-west) and 124% in Mkuranga (coast). Yield benefits of the new material were retained up to the final season in each location. The new variety (*Mkombozi*) introduced under community phytosanitation conditions in Chato yielded 86% more than the same variety from the same source planted in the no-phytosanitation control location. Although there was an 81% reduction in CBSD incidence in the new variety *Kiroba* introduced under community phytosanitation compared to control conditions in Mkuranga, there was no concomitant yield increase. Variety *Kiroba* is known to be tolerant to the effects of CBSD, and tuberous roots of infected plants are frequently asymptomatic. Community phytosanitation has the potential to deliver area-wide and sustained reductions in the incidence of CBSD, which also provide significant productivity gains for growers, particularly where introduced varieties do not have high levels of resistant/tolerance to CBSD. The approach should therefore be considered as a potential component for integrated cassava virus management programmes, particularly where new cassava plantations are being established in areas severely affected by CBSD.

## Highlights

• This study comprised the first attempt to manage cassava viruses with community phytosanitation for more than 70 years.
• Community phytosanitation resulted in area-wide reductions in CBSD incidence sustained over the 3-year duration of the study.
• Reduced CBSD and increased yields mean that community phytosanitation could be an important component of integrated cassava virus management.

## 1 Introduction

Cassava brown streak disease (CBSD) is one of the most important constraints to the production of cassava in Africa. Its continental significance has increased since the early part of the 21st century as new outbreaks were reported from the Great Lakes region of East and Central Africa (Alicai et al., 2007, Legg et al., 2011). Because of these new epidemics, concern has been raised about the potential for further westwards spread towards the major production zones of West-Central and West Africa (Legg et al., 2014a). Significantly, Nigeria is currently the world’s largest producer of cassava (FAOSTAT, 2017), and if CBSD was to spread there, its effects on cassava production could have a global impact.

Cassava brown streak disease is caused by two species of cassava brown streak ipomovirus (CBSI) (Family *Potyviridae*: Genus *Ipomovirus*): *Cassava brown streak virus* (CBSV) and *Ugandan cassava brown streak virus* (UCBSV) (Mbanzibwa et al., 2009). These are spread through stem cuttings when infected plants are used as a source of planting material, and they are also spread in a semi-persistent manner by the whitefly vector, *Bemisia tabaci* (Genn.) (Maruthi et al., 2005, Jeremiah, 2012). For many years, following the first report of CBSD in Tanzania (Storey, 1936), the geographical distribution of the disease was confined to coastal East Africa and the shores of Lake Malawi (Nichols, 1950). Recent epidemics reported from the Great Lakes region from 2004 onwards have been linked with the occurrence of super-abundant populations of the whitefly vector in these regions (Legg et al., 2011, Legg et al., 2014b, Legg et al., 2015). This change in the vector dynamics was considered to be the key factor in the earlier development and spread of the severe pandemic of cassava mosaic disease (CMD) (Otim-Nape et al., 1997), caused by cassava mosaic begomoviruses (CMBs), which are also transmitted by *B. tabaci* (Chant, 1958, Dubern, 1994). Although *B. tabaci* genotypes occurring on cassava may also colonize other host plants (Sseruwagi et al., 2006), their abundance on these hosts is so low as to render them insignificant in the epidemiology of the CBSIs and CMBs. Additionally, cassava is the only commonly-occurring host of the CBSIs. One alternative host is recognized in the published literature (*Manihot glaziovii* Muell.-Arg.) (Mbanzibwa et al., 2011), but this occurs infrequently in most areas of cassava cultivation.

Management efforts for both CBSD and CMD have mainly focused on the development of resistant varieties (Dixon et al., 2003, Kawuki et al., 2016). CMD-resistant varieties have been bred using conventional approaches, and these varieties are widely distributed through the cassava-growing regions of Africa (Manyong et al., 2000). By contrast, there has been less success with efforts to identify, introgress and disseminate sources of resistance to CBSD.

Phytosanitary measures have also been widely advocated for CMD and CBSD control. At farm-level, the removal of infected plants (roguing) and selection of healthy stems for replanting have frequently been recommended by research and extension agencies (Hillocks and Jennings, 2003). Recent studies, however, have highlighted important contrasts in the epidemiologies of the two virus groups causing these diseases. Persistently-transmitted CMBs are retained by the whitefly vector for much longer periods than CBSIs, allowing for longer distance spread (Dubern, 1994, Legg et al., 2011, Jeremiah, 2012). In addition, the symptoms of CBSD are much less readily recognized than those of CMD, since CMD is typically most severe on young leaves at the top of the plant, whilst CBSD symptoms are most commonly expressed on lower leaves, do not distort the leaf shape, and may readily be confused with other conditions such as mineral deficiency or leaf senescence (Nichols, 1950). Limited progress in developing CBSD resistance, the relative difficulty of recognizing CBSD symptoms in infected plants, and inefficiency of long-distance spread of CBSIs by the whitefly vector, together mean that there is an urgent need to control the disease through the implementation of a ‘clean seed’ programme. Systems for the production of healthy planting material have been initiated in several countries of East Africa worst affected by CBSD (IITA, 2014). These aim to ‘prime’ the system with virus-indexed plantlets produced and multiplied through tissue culture. This approach is combined with pre-basic field production of planting material in isolated locations within production zones, and a rigorous and formalized mechanism for inspecting and certifying seed production sites at pre-basic, basic and certified levels. Pre-basic cassava ‘seed’ (= planting material) is the highest quality ‘seed’, produced by breeders of the national research system. Basic ‘seed’ is produced by private seed companies or large farmers who receive pre-basic ‘seed’, multiply it and sell to certified ‘seed’ producers, who are large or medium-scale farmers. Cassava ‘seed’ at each of these levels is required to be inspected by national seed inspection authorities prior to being sold or distributed through formal seed dissemination programmes. As stocks of certified planting material at the various levels have become more readily available, questions have arisen about the best model through which to introduce certified planting material to village communities. Theoretically, since CBSIs spread poorly over distance, there should be a significant benefit to growers in reducing area-wide inoculum levels if they introduce healthy planting material as a group. In addition, the benefit of this approach should be greatly enhanced if existing sources of inoculum are removed prior to the introduction of clean planting material of the new variety. There is a precedent for community-level action of this type. In the 1930 and 1940s, epidemics of CMD had a devastating impact on cassava production through large parts of Uganda. This was addressed by a colonial government programme which enforced the removal of existing fields that were infected, and provided for their replacement with disease-free stocks of planting material with higher levels of CMD resistance (Jameson, 1964). The effort proved effective, and CMD was subsequently considered to be a disease of only moderate importance until the devastating epidemic that affected virtually all cassava-growing regions of the country in the 1980s and 1990s (Otim-Nape et al., 1997). There have been no subsequent efforts anywhere where CMD occurs in Africa or Asia to apply community-wide phytosanitation measures. This is partly a consequence of the relative success achieved in breeding for resistance, but also the difficulty of implementing such a programme under the less authoritarian community governance conditions of post-independence Africa. The contrasting characteristics of CBSD’s symptomatology and epidemiology, however, provided an opportunity to test a new community-level phytosanitation initiative which was the first of its kind for CBSD and the first for any cassava disease since the 1940s. The objective of this study was to determine if selected communities in regions affected by CBSD would be able to co-operate in applying phytosanitation to manage CBSD. More specifically, the study aimed to determine whether collective action involving groups of neighbouring cassava producers jointly applying phytosanitation measures would deliver improved disease management and yield outcomes in comparison with control communities in which material from the same source was introduced to single dispersed individuals who did not apply phytosanitary controls. The study was conducted between 2013 and 2016 in two communities in Tanzania, one in the north-west of the country next to Lake Victoria, and the second in the eastern coastal zone of the country. It was anticipated that if the results obtained were favourable, the approach might be scaled out more widely for community management of CBSD in all affected parts of East and Central Africa.

## 2 Materials and methods

### 2.1 Study location

**Fig. 1:**
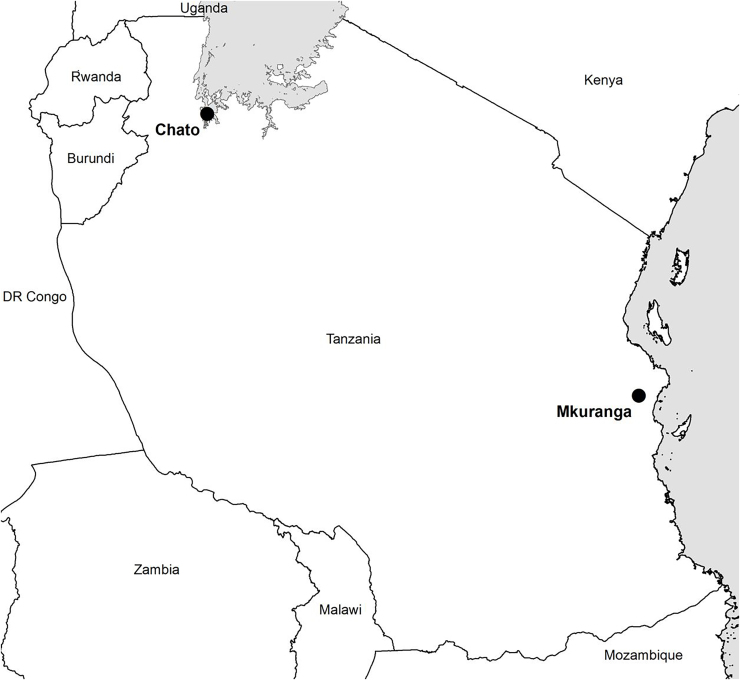
Location of community phytosanitation sites in Tanzania.

Four communities were initially targeted for the piloting of community phytosanitation in Tanzania. Two of these were in the Lake (Victoria) Zone of the north-west (in Muleba and Chato Districts) and two in the eastern Coast Zone (in Kisarawe and Mkuranga Districts). Following preliminary interactions with all communities, the programme determined to complete the initiative in one location per Zone: Chato in the Lake Zone and Mkuranga in the Coast Zone. These two locations were selected based on the importance of the cassava crop to the communities and the relative severity of CBSD. Mkuranga District is in the Coast Region, immediately to the south of the metropolitan Region of Dar es Salaam. Chato District is in Geita Region, on the shores of the south-west corner of Lake Victoria (Fig. 1).

### 2.2 Community sensitization, area-wide roguing and the phytosanitation strategy

Prior to the removal of existing plantings of cassava from the target communities, a year-long period of sensitization was conducted by the agencies working with farmers, including the research institutions, non-governmental organizations and extension service. Local leaders played a key role in raising awareness throughout their communities of the planned initiative. Community phytosanitation plans were explained to the community, including the essential elements of the technical justification and potential gains to be achieved. The proposed approach was to initiate the programme in one corner of the target community, through removal of all existing cassava plants, and immediately after this, to introduce certified sources of disease-free planting material of an improved variety with a moderate level of CBSD resistance. In the second season (where each season lasted for a period of one year), it was planned to extend the area covered to a larger portion of the community, and subsequently to continue in this way until the entire community had removed existing sources of infected planting material, and replaced them with new disease-free material (protocol for the sourcing of disease-free planting material described below). Both communities agreed to work together in the implementation of the programme, recognizing the importance of the goal of improving cassava production through reducing the impacts of cassava virus diseases.

The first part of the community to replace their cassava with disease-free planting material was designated the Primary Recipient Group (PRG). In the second year, the programme was extended to a Secondary Recipient Group (SRG), followed by a Tertiary Recipient Group (TRG) in the third year. Data were collected for all three stages in Chato, but for only the PRG and SRG stages in Mkuranga. In order to provide a control for the phytosanitation experiment, a neighbouring community was identified in both locations. A group of farmers in this community received disease-free planting material from the same source as farmers in the other groups (PRG, SRG and TRG), but these farmers were randomly dispersed through their community and did not remove any diseased plants − as is the custom for cassava farmers in Tanzania. This group was designated the Control Recipient Group (CRG).

Cassava plants of diseased local varieties were harvested and the stems destroyed during the final quarter of 2013 for the PRG in Chato, whilst this was done in the final quarter of 2014 for the PRG in Mkuranga. For each group of farmers (PRG, SRG, TRG), local infected plants were completely removed from the farming area prior to the introduction and planting of new disease-free material. In Chato, the new variety introduced was *Mkombozi*, which is highly resistant to CMD but moderately susceptible to CBSD. In Mkuranga, the introduced variety was *Kiroba*, which is moderately resistant to CMD but highly tolerant to CBSD. Both varieties have been officially released in Tanzania. *Mkombozi* (MM96/4684) was a CMD-resistant selection introduced to Tanzania under open quarantine in the late 1990s from the IITA regional cassava breeding programme based in Uganda. It was officially released in Tanzania in 2009. *Kiroba* is a local cultivar that was selected, evaluated, released (in 1998) and subsequently promoted by the national research system in Tanzania for the coastal parts of the country. Whilst *Kiroba* may be readily infected by CBSIs, and express symptoms of CBSD in its leaves, there is very little root damage. There was a limited amount of sharing of planting material from PRG to SRG, and from SRG to TRG following harvesting at each stage. However, for the comparison between the CRG and TRG in Chato (planted in November 2016) and the CRG and SRG in Mkuranga (planted in January 2016), disease-free planting material was obtained from identical sources for each location. Sources of disease-free planting material were the certified pre-basic cassava planting material production sites of Mtopwa, Mtwara Region (south-eastern Tanzania) and Kitengule, Kagera (north-western Tanzania). Certification included a virus testing protocol for CBSIs involving the testing of 200 plants per hectare and an assurance that the level of infection from CBSIs was less than 2%, coupled with a symptom incidence tolerance level of less than 1%. It is important to note, therefore, that whilst cassava planting material was supplied to communities as disease-free, it is possible that a small proportion of cuttings introduced were infected. This possibility was considered to be unavoidable, since both north-western and eastern coastal Tanzania are now considered to be endemic zones for CBSD.

For farmer groups where community phytosanitation was implemented, local task forces were established, comprising extension workers and farmer representatives, whose role it was to ensure that farmers in the group removed any introduced plants expressing symptoms of CBSD during the growing period. Similarly, task forces had the role of ensuring that farmers in these groups did not plant plots of local varieties, already recognized from the outset to be heavily infested by CBSD.

### 2.3 Farmer group disease monitoring

Ten farmers were selected at random from within each of the PRG, SRG, TRG and CRG groups at each of the two locations. Permission was sought from these farmers to monitor their fields, from planting through to harvest. Farmers’ own management practices were applied, including planting on ridges and manual weeding with a hand hoe (typically after sprouting and two to three times subsequently). No fertilizer applications were made to any of the plots, following usual farmer practice in Tanzania. In each of the monitored fields, the data described below were collected at three to four evenly-spaced intervals throughout the growing season, starting from two months after planting (2MAP). One hundred plants were assessed along two diagonals running across the field, in the form of an ‘X’.

#### 2.3.1 *Bemisia tabaci* whiteflies

Adult whiteflies were counted manually using standard procedures (Sseruwagi et al., 2004) on the top five apical leaves of the tallest shoot of each of 100 plants assessed. Total numbers on the five top leaves were calculated for each sampled plant, and these values were averaged to produce a mean whitefly abundance for that assessment.

#### 2.3.2 Cassava brown streak disease (CBSD)

Leaf and shoot symptoms were assessed using a severity scale of 1–5 in which 1 represented no symptoms and 5 represented the most severe symptoms that included stem streaking and shoot tip die-back (Hillocks and Thresh, 2000). Incidence was calculated as the percentage of symptomatic plants.

#### 2.3.3 Harvest data

Monitored plots were harvested from 9 to 10 MAP. In each field, five blocks of 20 plants each (total of 100 plants) were uprooted for the measurement of harvest data. Each block was selected at random, and plants measured excluded the border row plants. The data described below were recorded for each of the harvested plants. A small number of the CRG fields in Chato and Mkuranga were harvested by growers prior to the final planned harvest date and were therefore not represented in the yield comparisons.

##### 2.3.3.1 Harvested area

The harvested area was calculated by multiplying the length and width of each harvested block. Dimensions were measured using a tape measure and included an extension of 0.5 m beyond the outermost harvested plant, since the plant–plant spacing was typically 1 m x 1m.

##### 2.3.3.2 Tuberous root weight

The weight of each tuberous root was measured using a field weighing balance. Total root weight harvested was calculated for each block, and this was divided by the harvested area to give an estimate of yield per unit area. This figure was then converted to a t/ha value. The weight of unusable roots, defined below, was not subtracted from the gross yield figure.

##### 2.3.3.3 CBSD root symptoms

Each root was cut in cross-section five times to produce evenly-sized slices. The first cut was made at the tip of the tuberous root, and the final cut was made closest to the point at which it was attached to the stem. The severity of CBSD symptoms for each slice was recorded using a severity scale of 1–5, in which 1 represented no root necrosis and 5 represented the most severe necrosis. This was based on the scoring system of Hillocks and Thresh (2000).

##### 2.3.3.4 CBSD foliar symptoms

CBSD foliar symptoms, and presence or absence of stem symptoms were recorded for each harvested plant. The assessment of foliar symptoms was based on the severity scale of 1–5 (Hillocks and Thresh, 2000). For stem symptoms, ‘+’ was used to indicate present and ‘−’ for absent.

##### 2.3.3.5 CBSD incidence values recorded at harvest

Four incidence values were obtained following harvest, following the approach of Ndyetabula et al. (2016). These were:

• Plant total incidence. The percentage of harvested plants expressing any symptoms of CBSD, in either the foliar part of the plant or the roots.
• Plant shoot incidence. The percentage of harvested plants expressing foliar CBSD symptoms
• Plant root incidence. The percentage of harvested plants expressing CBSD root symptoms in one or more roots.
• Root incidence. The percentage of roots with symptoms of CBSD.
• Unusable root incidence
• The percentage of roots where at least one of the five cross-sectional cuts had a symptom score of “3” or more.

#### 2.3.4 Replanting

Following harvest, disease-free stems were selected from amongst the harvested plants and used to re-plant the experiment. In Chato, PRG fields were replanted for two consecutive seasons at the same PRG location, giving a total of three monitored plantings (PRG-season 1, PRG-season 2 and PRG-season 3, each of one year duration). SRG farmers that were monitored received near virus-free planting material from the clean ‘seed’ source in the second year of the programme (SRG-season 1), and they replanted once in year 3 (SRG-season 2). Fields of the CRG and TRG groups were planted once, in year 3. After the first year’s plantings at Mkuranga (PRG-season 1), PRG fields were replanted once (PRG-season 2), whilst SRG and CRG fields were not replanted.

### 2.4 Local variety comparisons

In Chato, at the time that each of the PRG, SRG and TRG groups were initiated, assessments were made for the same number of fields of local varieties at harvest age. Yield was determined in these fields using the same method employed for the PRG, SRG and TRG fields. These fields were referred to as ‘baseline’ fields, as they provided a measure of yields obtained by farmers with their own varieties prior to the implementation of the community phytosanitation approach. The same approach was used to determine baseline yields for PRG and SRG groups in Mkuranga. In addition, at the same time that PRG field monitoring began in Chato and Mkuranga, a set of ten fields planted with local varieties in the SRG location was monitored. This allowed for a season-long comparison of patterns of virus disease development in the PRG and local variety fields (located in the SRG area that had not yet started to apply community phytosanitation). These fields were referred to as ‘local’ fields. Baseline fields were only assessed once, at the time of harvest. ‘Local’ fields were assessed throughout the course of the season, using the same methods employed for monitoring the PRG, SRG, TRG and CRG fields.

### 2.5 CBSD inoculum pressure assessment

Inoculum pressure was assessed for monitored fields in both Chato and Mkuranga in the first quarter of 2016. This involved the determination of the incidence of CBSD in all fields within a 250 m radius of monitored fields within the PRG, SRG, TRG and CRG groups together with the estimation of the distance between surrounding and monitored fields. Georeferenced locations were obtained for all cassava fields using a GPS unit, and this facilitated the calculation of distances between surrounding and monitored fields using ArcGIS software (ArcGIS 10.1, ESRI, California, USA). Three measures were used as an estimate of inoculum pressure:

• Surrounding CBSD. Calculated as the sum of inoculum pressure values for each of the fields surrounding the respective monitored field, where each inoculum pressure value was the number of CBSD-infected plants in the surrounding field divided by the distance from the monitored field. The number of CBSD-infected plants was determined by sampling 100 plants to calculate the infected proportion, then multiplying this by the estimated plant population.
• Surrounding CBSD Inoculum Index 1 (Sur 1). Indices were calculated for mean whitefly abundance in each surrounding field, where the maximum abundance value was transformed to 1 and the minimum to zero. Indices were calculated in a similar way for the number of plants infected by CBSD. *Sur 1* was calculated as the average of the whitefly and CBSD index values for each surrounding field.
• Surrounding CBSD Inoculum Index 2 (Sur 2). *Sur 2* was calculated similarly to *Sur 1*, with the exception that natural log values for both whitefly abundance and CBSD infected plant number were used when calculating the index values.

### 2.6 GIS and data analysis

Values for CBSD incidence and Sur 1 were extrapolated from point to surface level using ordinary kriging (Goovaerts, 1997). The technique of kriging predicts the value of a variable at an unmeasured location from observed values at surrounding locations. We used the default settings of the geostatistical module in ArcGIS to determine the nugget, the range, the number of lags and the lag-size. These default values are different per variable as the statistical properties of the variables differ.

Standard error values for CBSD incidence and yield data were calculated in MS Excel, whilst ANOVA, *t*-tests and linear regression analyses were performed using the SigmaStat module of SigmaPlot 11.0 (Systat Inc., San Jose, USA).

## 3 Results

### 3.1 Implementation of phytosanitation

The initial removal of existing cassava fields in the PRG areas of both Chato and Mkuranga proceeded according to the plan agreed with the communities. Following the sensitization period, farmers were ready to comply with the requirement to harvest and remove plants in existing cassava fields, and taskforces in each of the two locations supervised the process effectively. Regular visits to the monitored PRG fields ensured that any plants expressing CBSD symptoms were rogued. This procedure was less effectively applied in the PRG fields that were not regularly monitored, and where roguing was to be supervised by the taskforces. During the expansion of the process to include SRG areas and monitored SRG groups, there was significant resistance in Chato to the requirement to harvest and remove existing fields. The SRG covered a much larger area than the PRG, there was a larger proportion of commercial farmers who had made cash payments for cultivation, and several of the fields were at an early stage of development and not ready for harvest. Compensation measures were reinforced, including the provision of free maize seed and sweet potato planting material, and the process was completed. Over the course of the programme there was a gradual but significant re-introduction of small quantities of local varieties, particularly in the PRG and SRG locations of Chato. During the assessment of fields surrounding monitored PRG fields in 2016, 38.2% of these fields had at least some local variety plants present. For SRG fields at the same time, the value was 61.4%. Re-introduction of plants of local varieties was less significant in Mkuranga.

### 3.2 CBSD incidence

#### 3.2.1 Baseline assessments

The baseline assessments of CBSD in the existing local variety fields of PRG and SRG farmers in Chato, prior to the implementation of the community phytosanitation approach, revealed high levels of CBSD. For the PRG zone, the incidence of plants affected by CBSD was 93.0%, although the unusable root incidence was lower at 4.5% (Table 1). For Chato SRG farmers whose plots were harvested in the following year, the overall incidence of plants affected was 97.3% and 5.8% of roots had severe damage (Table 2), whilst for the TRG group, the overall incidence of infected plants was 98.3% and the incidence of unusable roots 12.7% (Table 3). These data confirm the high level of CBSD incidence in local varieties being cultivated by farmers prior to the intervention.

**Table 1:** CBSD incidences and yield for PRG baseline fields with local varieties − Chato.

Farmer	Plant total incidence^a^	Plant shoot incidence^b^	Plant root incidence^c^	Root incidence^d^	Unusable root incidence^e^	Yield^f^ t/ha
Ayubu Mtangi	61.0	58.0	8.0	3.7	0.6	8.9
Bryson Charles	100	100	25.0	11.5	3.3	9.2
Bujumbura Daideli	97.0	96.0	38.0	16.0	6.4	9.5
Ndebile Balitazari	90.0	90.0	14.0	11.3	4.9	1.1
Helena Segera	96.0	93.0	14.0	3.8	2.2	5.8
James Mtangi	96.0	96.0	17.0	8.7	2.3	3.2
John Bilingi	97.0	97.0	14.0	2.5	2.2	3.8
Helena Munyankore	98.0	94.0	27.0	13.8	12.5	2.2
Ester Simon	97.0	97.0	4.0	1.9	1.9	1.4
Priscus Severin	98.0	93.0	35.0	14.2	9.8	7.6
Overall Mean	**93.0**	**91.4**	**19.5**	**8.7**	**4.5**	**5.2**

a **Plant total incidence** − Percentage of plants with CBSD symptoms in either shoots or roots.

b **Plant shoot incidence** − Percentage of plants with CBSD symptoms in shoots.

c **Plant root incidence** − Percentage of plants with CBSD symptoms in roots.

d **Root incidence** − Percentage of roots with CBSD symptoms.

e **Unusable root incidence** − Percentage of roots with at least one cut (out of five) with CBSD severity score 3 or more.

f **Yield** − Yield of tuberous roots harvested from five blocks of 20 plants per farmer’s field, measured in t/ha.

**Table 2:** CBSD incidences and yield for SRG baseline fields with local varieties − Chato.

Farmer	Plant total incidence^a^	Plant shoot incidence^b^	Plant root incidence^c^	Root incidence^d^	Unusable root incidence^e^	Yield^f^ t/ha
Marco Mussa	92.0	89.0	22.0	6.6	2.8	9.9
Pascal Mathew	100.0	96.0	24.0	9.8	5.1	4.5
Mathew Magazi	97.0	93.0	24.0	7.2	4.3	6.6
Andrea Katigula	97.0	97.0	26.0	8.8	5.8	3.9
Bernard Kasamwa	99.0	99.0	63.0	27.3	11.4	5.8
Andrew Kasama	96.0	94.0	57.0	32.4	17.6	4.9
Buswetula Kisatu	96.0	96.0	20.0	8.0	2.6	7.3
Lucas Kamulajone	99.0	99.0	28.0	8.1	3.2	6.6
Mwakoye Mwanzalima	97.0	94.0	21.0	7.2	3.3	6.5
Emmanuel Philemmon	100	100	3.0	1.4	1.4	0.6
Overall Mean	**97.3**	**95.7**	**28.8**	**11.7**	**5.8**	**5.7**

a **Plant total incidence** − Percentage of plants with CBSD symptoms in either shoots or roots.

b **Plant shoot incidence** − Percentage of plants with CBSD symptoms in shoots.

c **Plant root incidence** − Percentage of plants with CBSD symptoms in roots.

d **Root incidence** − Percentage of roots with CBSD symptoms.

e **Unusable root incidence** − Percentage of roots with at least one cut (out of five) with CBSD severity score 3 or more.

f **Yield** − Yield of tuberous roots harvested from five blocks of 20 plants per farmer’s field, measured in t/ha.

**Table 3:** CBSD incidences and yield for TRG baseline fields with local varieties − Chato.

Farmer	Plant total incidence^a^	Plant shoot incidence^b^	Plant root incidence^c^	Root incidence^d^	Unusable root incidence^e^	Yield^f^ t/ha
RozaLunyirija	100	100	43	12.8	13.2	4.0
Christina Marko	100	100	55	18.9	16.9	3.3
Boniface Masalu	100	100	41	18.5	16.4	3.9
Zakaria Kulwa	100	100	42	16.4	14.2	2.9
Shija Peter	100	100	35	22.6	20.1	2.5
Antony Robert	100	100	18	10.1	9.3	1.9
MegejiwaDoya	92	90	18	8.3	7.4	2.5
Thomas William	97	97	38	18.1	10.7	2.2
Jacob Andrew	96	96	21	14.5	11.7	2.1
Welima Justine	98	98	14	7.8	6.9	1.5
Overall Mean	**98.3**	**98.1**	**32.5**	**14.8**	**12.7**	**2.7**

a **Plant total incidence** − Percentage of plants with CBSD symptoms in either shoots or roots.

b **Plant shoot incidence** − Percentage of plants with CBSD symptoms in shoots.

c **Plant root incidence** − Percentage of plants with CBSD symptoms in roots.

d **Root incidence** − Percentage of roots with CBSD symptoms.

e **Unusable root incidence** − Percentage of roots with at least one cut (out of five) with CBSD severity score 3 or more.

f **Yield** − Yield of tuberous roots harvested from five blocks of 20 plants per farmer’s field, measured in t/ha.

#### 3.2.2 CBSD incidence in Chato

**Fig. 2:**
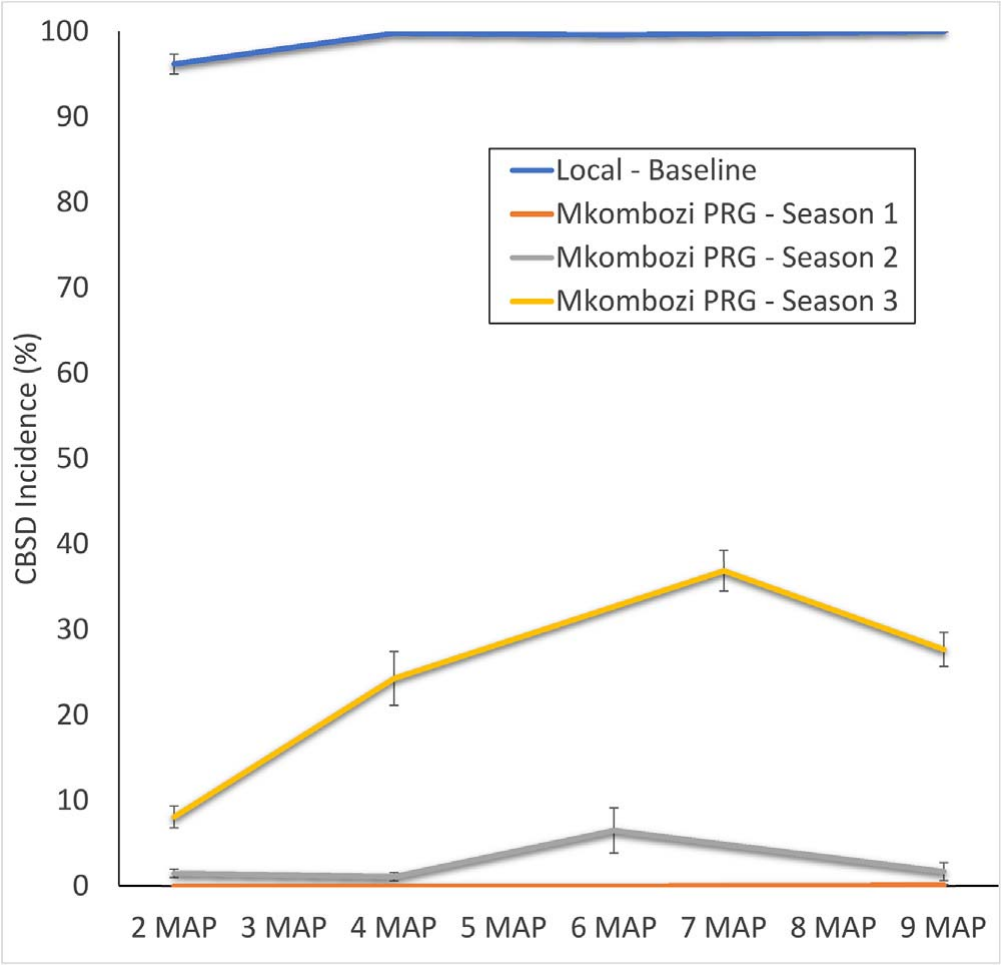
CBSD disease progress curves for three seasons in the Primary Recipient Group (PRG), Chato. Data from three successive seasons in which PRG farmers grew initially disease-free variety *Mkombozi* are compared with CBSD progress in the local variety comparator. Local variety data were recorded in season 1.

**Fig. 3:**
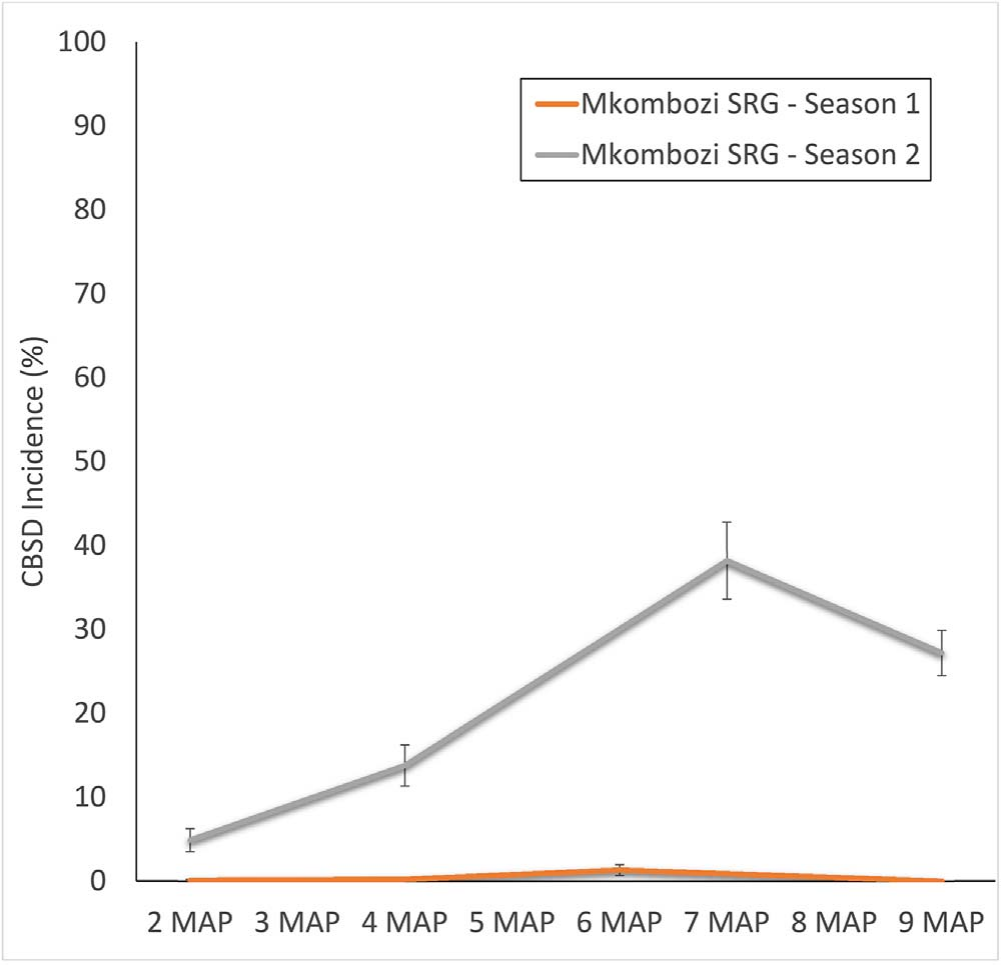
CBSD disease progress curves for two successive seasons in the Secondary Recipient Group (SRG), Chato, in which farmers grew initially disease-free variety *Mkombozi*.

**Fig. 4:**
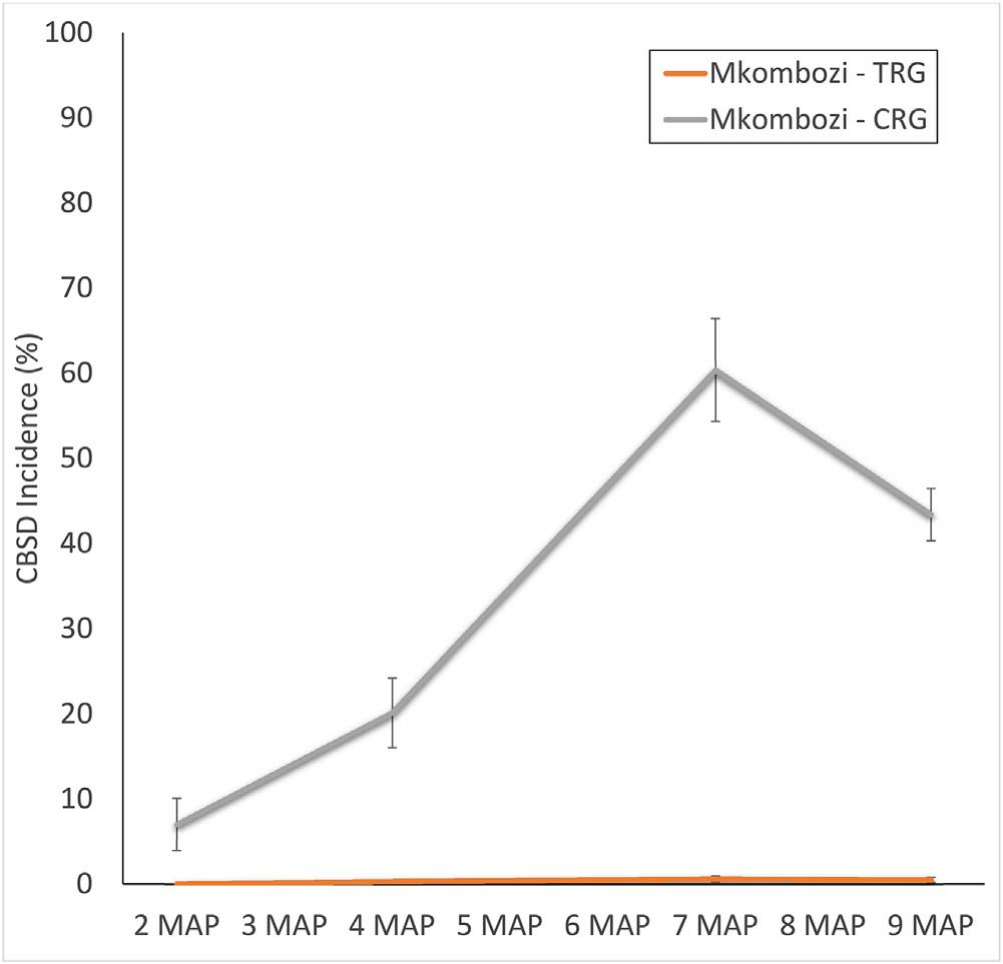
Comparison of CBSD disease progress curves for plots of variety *Mkombozi* grown by farmers in the Tertiary Recipient Group (TRG), Chato, who practised community phytosanitation and farmers of the neighbouring Control Recipient Group (CRG), who did not.

Disease progress curves for CBSD (Fig. 2, Fig. 3, Fig. 4) illustrate the progression of the disease in both local varieties and the introduced disease-free *Mkombozi* for each of the monitored farmer groups in Chato. Over the course of three consecutive seasons, CBSD incidence increased in the monitored plots of the PRG from 0 (lowest incidence, in season 1) to 36.9% (highest incidence, in season 3), although the final incidence at season 3 harvest was 27.7% (Fig. 2). Final CBSD incidences for the first and second seasons in the PRG group were 0.1% and 1.7% respectively. Some reductions in incidence within community phytosanitation plots between monitoring events occurred since some effort was made to remove symptomatic plants. The local variety field that was monitored concurrently with the first PRG planting, contrastingly had an initial incidence of 96.2% and by harvest all monitored plants were infected.

For monitored SRG fields, the final incidence of the first season’s planting was 0%, although this rose to a final incidence of 27.2% in the re-planted crop of the second season (Fig. 3). TRG fields allowed for a comparison between disease-free *Mkombozi* managed with community phytosanitation and the unmanaged control of the CRG (Fig. 4). After a single season, CBSD incidence in the TRG was 0.5%, whilst that in the CRG was 43.4%.

#### 3.2.3 CBSD incidence in Mkuranga

**Fig. 5:**
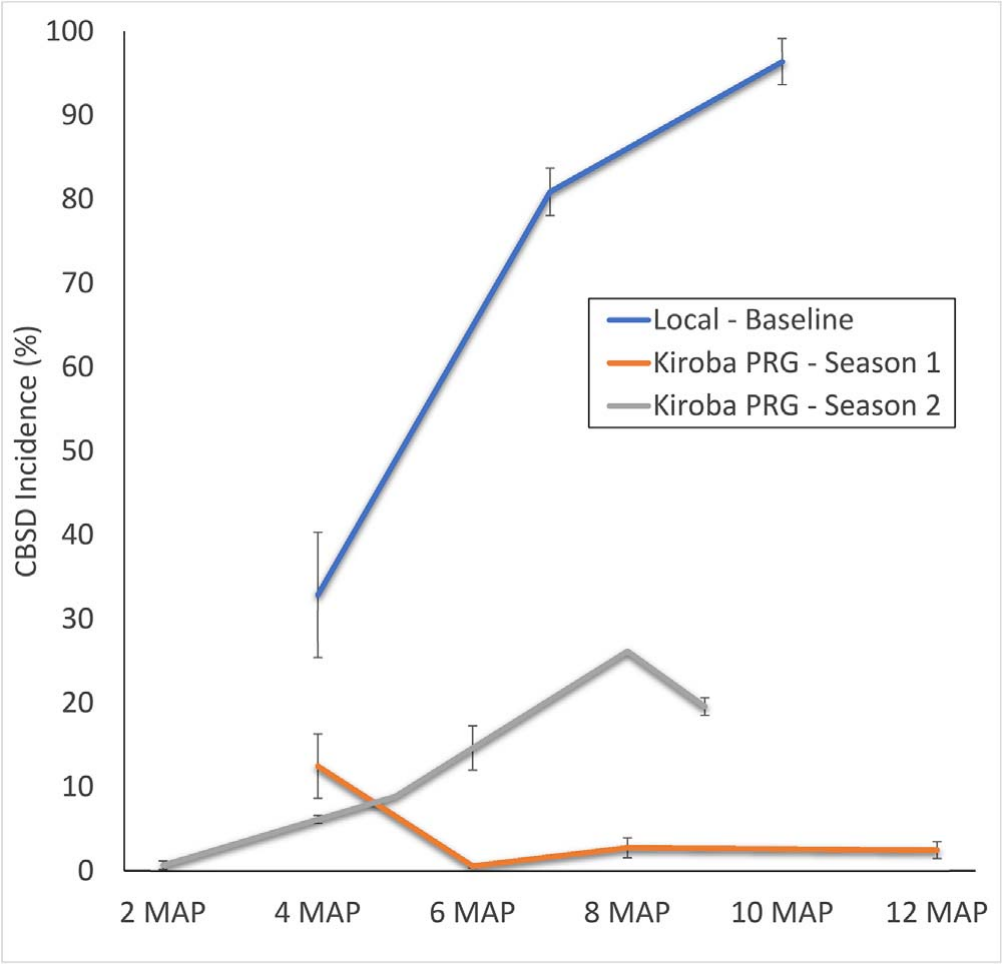
CBSD disease progress curves for two seasons in the Primary Recipient Group (PRG), Mkuranga. Data from two successive seasons in which PRG farmers grew initially disease-free variety *Kiroba* are compared with CBSD progress in the local variety comparator. Local variety data were recorded in season 1.

Incidence of CBSD in the monitored fields of the local variety increased from 32.9% to a final value of 96.4% at harvest (Fig. 5). The incidence in initially disease-free *Kiroba* in the first season of the PRG was 12.5% at 4 MAP, but declined to 2.5% at harvest due to roguing implemented through the community phytosanitation approach.

**Fig. 6:**
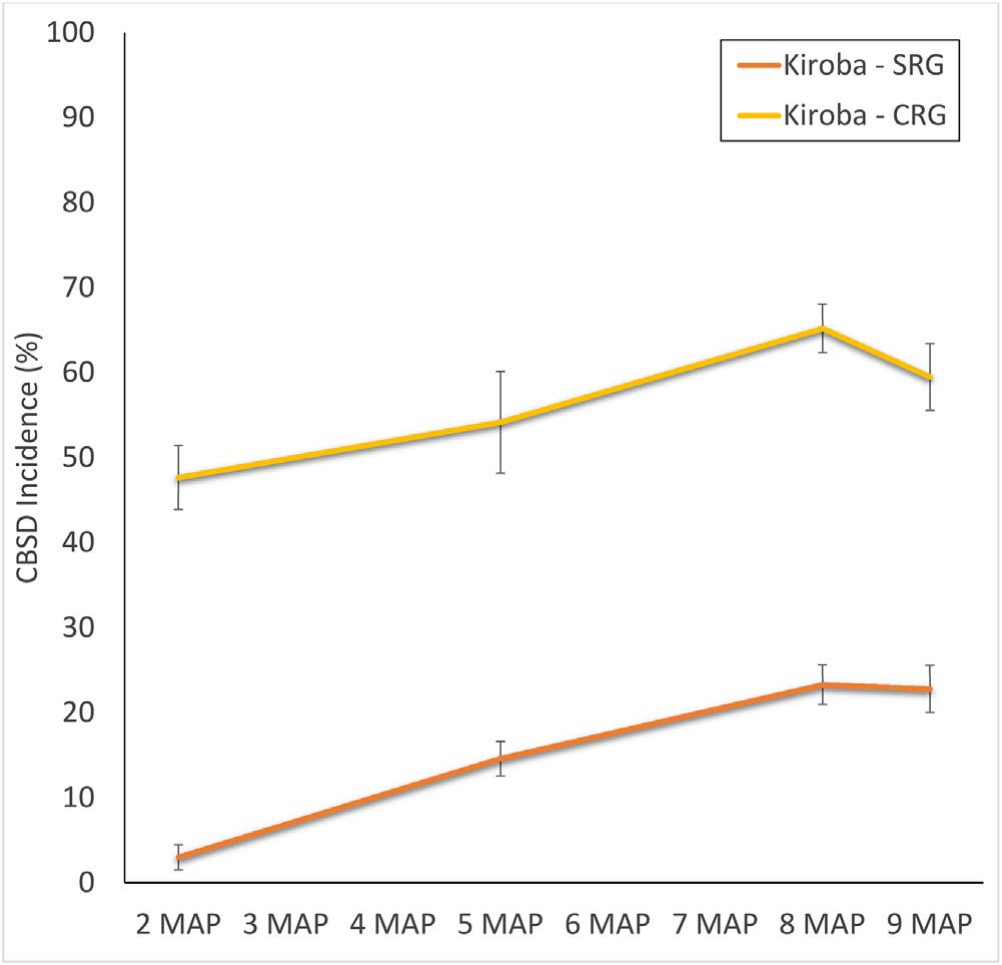
Comparison of CBSD disease progress curves for plots of variety *Kiroba* grown by farmers in the Secondary Recipient Group (SRG), Mkuranga, who practised community phytosanitation and farmers of the neighbouring Control Recipient Group (CRG), who did not.

Incidence in the SRG increased to 22.8% in the final recording, in comparison to 59.5% for CRG fields (Fig. 6). Although both plantings used cuttings derived from the same disease-free source of *Kiroba*, CRG plots were rapidly infected shortly after sprouting.

#### 3.2.4 Harvested yield in Chato

**Fig. 7:**
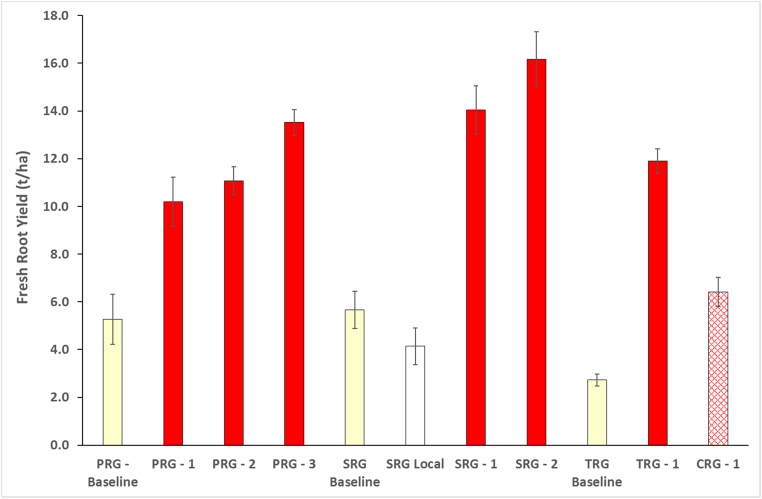
Fresh root yields recorded from farmer groups in Chato for PRG, SRG, TRG and CRG plantings. Baseline yields were recorded in local varieties grown in PRG, SRG and TRG locations prior to the commencement of community phytosanitation and the introduction of disease-free *Mkombozi.* The SRG local fields were cultivated with local varieties and monitored in parallel with PRG *Mkombozi* fields prior to the start of community phytosanitation in the SRG area.

Yields for all plantings of initially disease-free *Mkombozi* at all sites (PRG, SRG, TRG) were greater than baseline yields of farmers’ local varieties and the monitored plantings of local varieties at the SRG site (Fig. 7). Significantly, the yield of *Mkombozi* in the community phytosanitation managed TRG site (11.9 t/ha) was greater (t = 6.47, *P <* 0.001) than that of *Mkombozi* in the CRG control site (6.4 t/ha). Although there were slight increases in CBSD incidence in the PRG and SRG monitored plots of *Mkombozi*, these were not associated with declines in yield. The highest PRG yield was obtained in the final season (13.5 t/ha) and the greatest yield for all Chato plantings was obtained from the final SRG season (16.2 t/ha).

#### 3.2.5 Harvested yield in Mkuranga

**Fig. 8:**
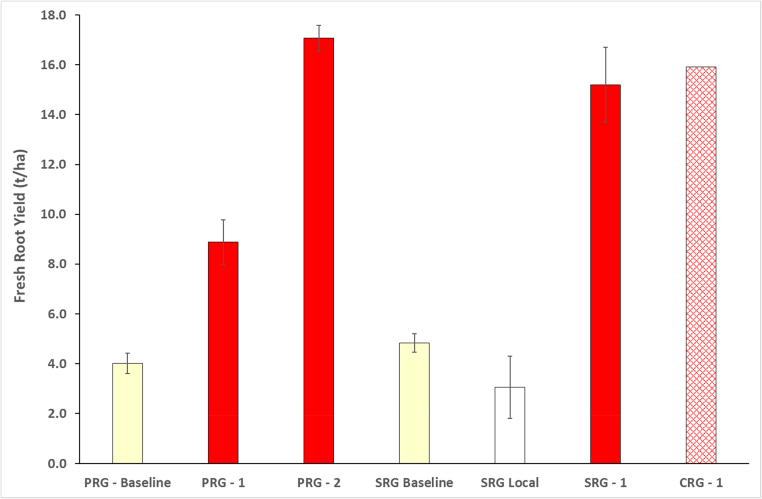
Fresh root yields recorded from farmer groups in Mkuranga for PRG, SRG and CRG plantings. The SRG local fields were cultivated with local varieties and monitored in parallel with PRG *Kiroba* fields prior to the start of community phytosanitation in the SRG area.

All yields for plots planted with *Kiroba* were greater than the PRG and SRG baselines, as well as the monitored local variety (Fig. 8). The mean yield of the second PRG planting was more than three times that of the local variety baseline, and the average SRG yield was more than three times that of the SRG baseline. As at Chato, there was no decline in yield recorded from the first to the second season of the PRG. In contrast to Chato, however, there was no significant difference in the yields of the SRG and CRG plots that were planted with cuttings of *Kiroba* obtained from the same source.

### 3.3 Whitefly abundance

#### 3.3.1 Chato

In all three seasons of the PRG, *B. tabaci* whiteflies were most abundant at the first (2MAP) recording. Numbers were greatest in the second season (33.1, S.E. ± 5.8) followed by the first (11.6, ± 2.3) and the third (7.1, ±1.1). The pattern of change in abundance over time was the same for the SRG, where highest abundances recorded at 2MAP were 66.4 ±9.5 (season 1) and 8.4 ±2.6 (season 2). Whiteflies were present in similar numbers on the monitored local varieties (12.0, ±3.5) when compared with concurrent PRG first season plantings. Maximum abundances observed at 2MAP in both the TRG and CRG groups were similar (TRG, 12.3 ±1.8; CRG 14.0 ±3.2).

#### 3.3.2 Mkuranga

The development of whitefly populations in Mkuranga generally followed a similar trend to those in Chato, as numbers were greatest during the early part of the season and declined thereafter. Whitefly abundance in the local monitored plots at the SRG site was greatest at 4MAP (15.4 ±2.2) and similar to the maximum abundance at 6MAP in the first season PRG plots of *Kiroba* (13.6 ±12.2). Abundance in the second season of the PRG was greatest at 2MAP (20.8 ±3.1), as it was in the CRG plots (94.4 ±30.2), although in this case the CRG whitefly abundance was significantly greater than that of the concurrent SRG plots (t = 3.2, *P* = 0.006).

### 3.4 Inoculum pressure

**Fig. 9:**
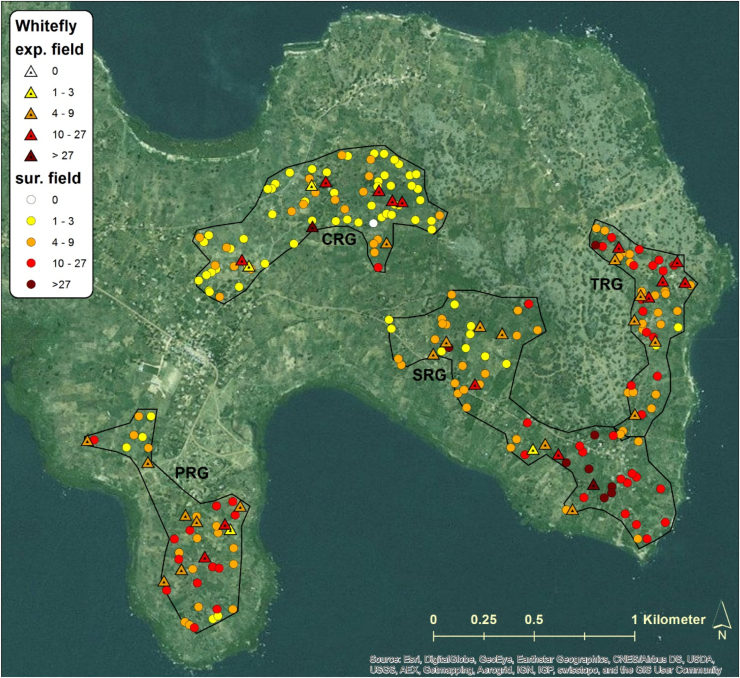
Whitefly abundance in experimental (monitored) and surrounding fields in the PRG, SRG, TRG and CRG areas of Chato, north-western Tanzania.

**Fig. 10:**
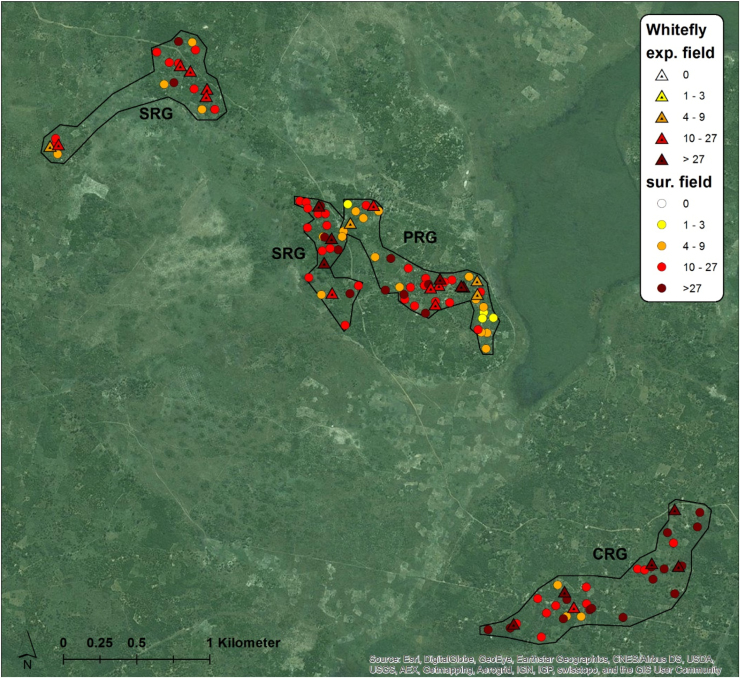
Whitefly abundance in experimental (monitored) and surrounding fields in the PRG, SRG, and CRG areas of Mkuranga, eastern coastal Tanzania.

**Fig. 11:**
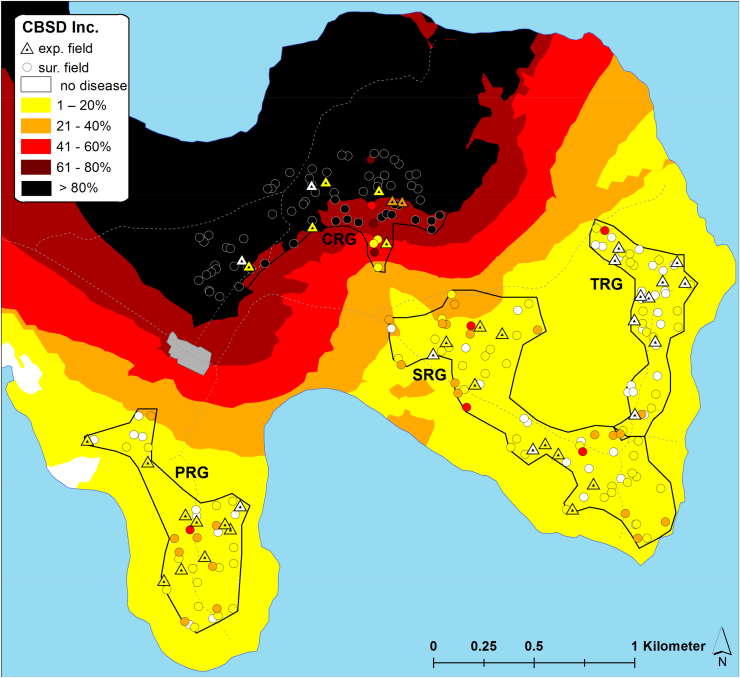
CBSD incidence in experimental (monitored) and surrounding fields in the PRG, SRG, TRG and CRG areas of Chato, north-western Tanzania. CBSD incidence data from all fields were used to develop an interpolated spatial plot of CBSD incidence using Kriging techniques.

**Fig. 12:**
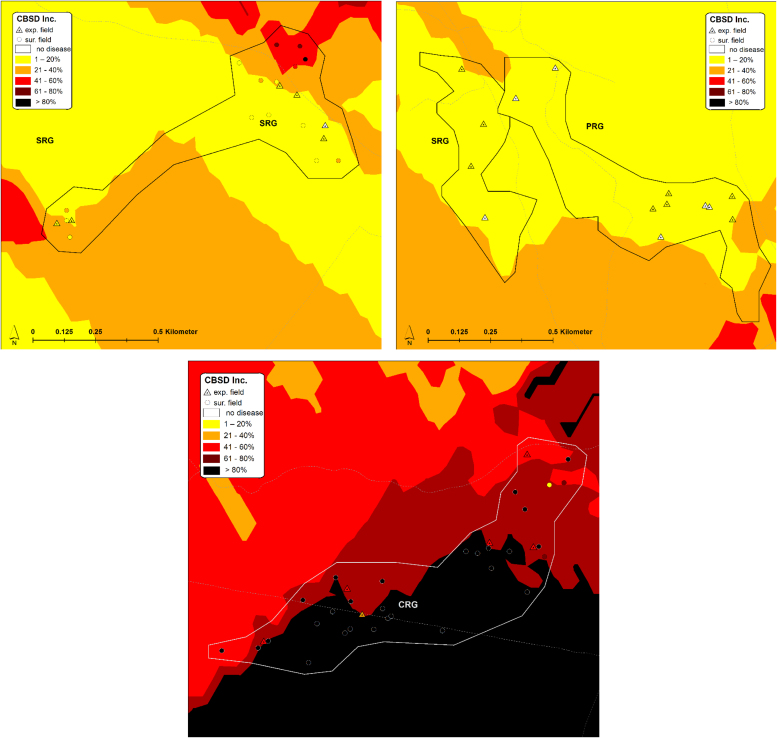
CBSD incidence in experimental (monitored) and surrounding fields in the PRG, SRG and CRG areas of Mkuranga, eastern coastal Tanzania. CBSD incidence data from all fields were used to develop an interpolated spatial plot of CBSD incidence using Kriging techniques.

**Fig. 13:**
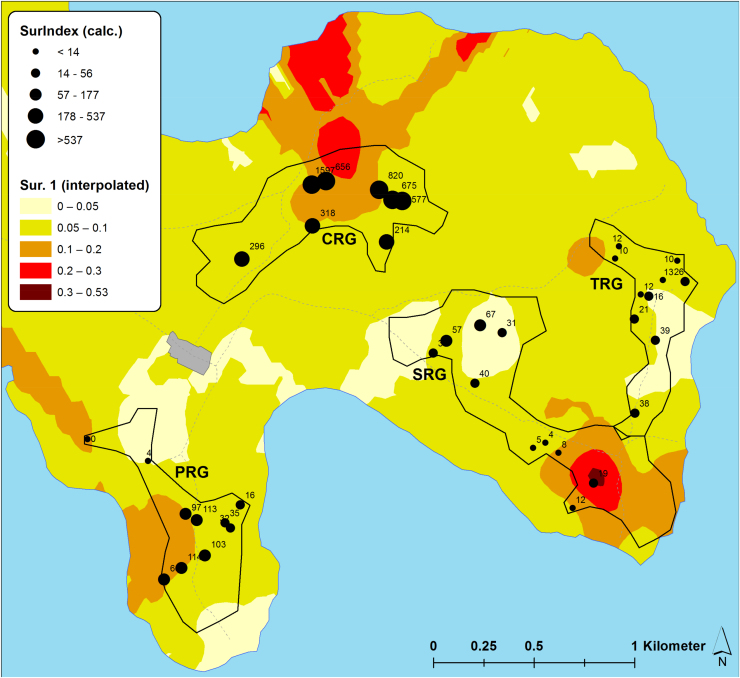
Surrounding CBSD values for PRG, SRG, TRG and CRG fields of Chato, north-western Tanzania, plotted over an interpolated spatial plot of Sur 1.

**Fig. 14:**
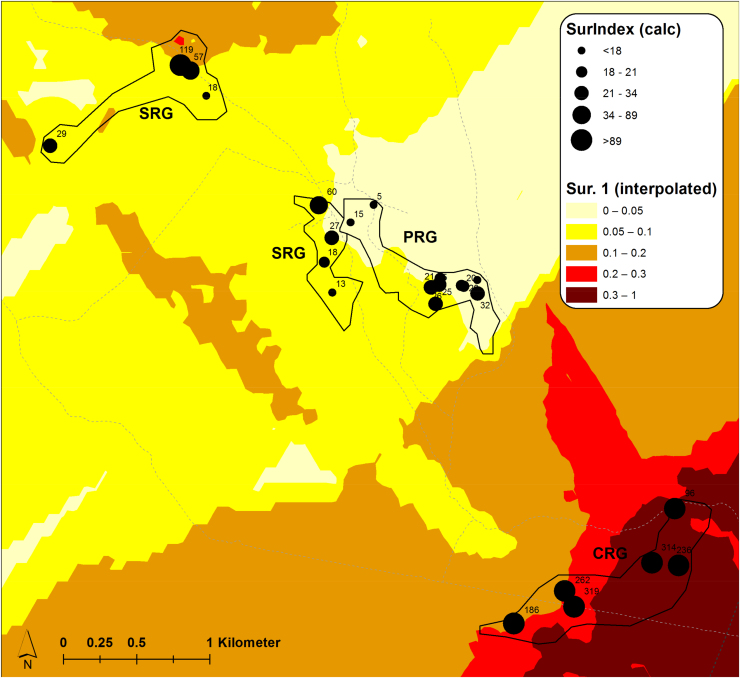
Surrounding CBSD values for PRG, SRG and CRG fields of Mkuranga, eastern coastal Tanzania, plotted over an interpolated spatial plot of Sur 1.

The inoculum pressure that is assumed to determine patterns of CBSD infection in initially CBSD-free cassava plantings was estimated based on measurements of CBSD incidence, plant number and whitefly abundance in fields surrounding the monitored plots. In this study, the estimate considered all cassava fields within a radius of 250 m from monitored plots and geo-referencing allowed distances between fields to be measured automatically with GIS software rather than manually using physical measurements. GIS maps illustrate the distribution of sites, the abundance of whiteflies in both monitored and surrounding fields, and the incidence of CBSD for both locations at the time of the inoculum pressure assessment in the first quarter of 2016 (Fig. 9, Fig. 10, Fig. 11, Fig. 12). The interpolated surfaces for CBSD incidence in Fig. 11, Fig. 12 highlight the strong gradient of CBSD between community phytosanitation sites (PRG, SRG and TRG) and the control sites (CRG). Assessments of Surrounding CBSD for each of the sites (Table 4; Fig. 13, Fig. 14) show that there were slightly more cassava fields surrounding the control sites than the community phytosanitation sites. A much stronger contrast was apparent, however, in the Surrounding CBSD values for CRG versus community phytosanitation sites: all CRG fields had higher Surrounding CBSD values than PRG, SRG or TRG sites from the same location. These results confirm the reduction in CBSD inoculum produced by the community phytosanitation interventions.

**Table 4:** Inoculum pressure assessed using surrounding CBSD values for community phytosanitation managed (PRG, SRG and TRG) and control fields of cassava in Chato and Mkuranga.

	N^a^	Min no. Surrounding Fields^b^	Max no. Surrounding Fields^c^	Min. Surrounding CBSD^d^	Max. Surrounding CBSD^e^	Ave. Surrounding CBSD^f^
Chato						
CRG	9	15	32	214.3	1596.6	625
PRG	10	3	15	0.29	114.1	57.9
SRG	10	7	18	4.2	66.8	28.1
TRG	10	14	26	9.8	39.3	19.8

Mkuranga						
CRG	6	5	12	95.7	319.3	141.4
PRG	10	5	19	5	31.8	20.5
SRG	10	3	17	7.7	73.7	34

a Number of cassava fields surrounding monitored plots (within a radius of 250m).

b Number of cassava fields surrounding the monitored plot with the fewest surrounding fields.

c Number of cassava fields surrounding the monitored plot with the most surrounding fields.

d Minimum surrounding CBSD value for monitored plots in this group.

e Maximum surrounding CBSD value for monitored plots in this group.

f Average surrounding CBSD value for monitored plots in this group.

In addition to the distance-based method for calculating CBSD inoculum pressure, surrounding CBSD indices were calculated for each of the monitored fields using a GIS kriging approach. Two measures of this index were compared: Sur1 and Sur2 (in which Sur2 used natural log transformations of CBSD incidence and whitefly abundance data). Preliminary tests indicated that Sur2 provided comparable results to those of Sur1, so final analyses used only Sur1. Linear regression analyses examining the relationship between Surrounding CBSD and Sur 1 produced strongly significant results for all monitored sites at Chato, Mkuranga, as well as the two sites combined (Table 5; Fig. 13, Fig. 14). When using the same variables as potential predictors for CBSD incidence in monitored fields (Table 5), predictions were significant for Mkuranga but not for Chato when considering CBSD incidence at 2MAP. Where Surrounding CBSD and Sur1 were used to predict future maximum incidences of CBSD in monitored fields (7MAP in Chato and 8MAP in Mkuranga), results were strongly significant for both sites. These results demonstrate that the measures of inoculum pressure used in this study were both strong predictors of CBSD incidence in monitored fields, and additionally, that both functioned similarly in this respect.

**Table 5:** Regression relationships between inoculum pressure variables and CBSD incidence.

X	Y	F	*P*	r^2^	Const.	m
a*Ch SurCBSD*	*Ch Sur 1*	*10.18*	*0.003*	*0.22*	*0.071*	*0.000036*
b*Mk SurCBSD*	*Mk Sur 1*	*73.83*	*<0.001*	*0.76*	*0.034*	*0.0010*
c*All SurCBSD*	*All Sur 1*	*4.85*	*0.031*	*0.073*	*0.080*	*0.000079*
*Ch SurCBSD*	*Ch CBSD 2MAP*	*0.85*	*0.36*	*0.023*	*0.042*	*0.000028*
*Mk SurCBSD*	*Mk CBSD 2MAP*	*64.76*	*<0.001*	*0.73*	*−0.0087*	*0.0018*
*All SurCBSD*	*All CBSD 2MAP*	*2.10*	*0.15*	*0.033*	*0.065*	*0.000099*
d*Ch Sur 1*	*Ch CBSD 2MAP*	*1.88*	*0.18*	*0.050*	*0.0058*	*0.54*
*Mk Sur 1*	*Mk CBSD 2MAP*	*104.40*	*<0.001*	*0.81*	*−0.053*	*1.62*
*All Sur 1*	*All CBSD 2MAP*	*157.63*	*<0.001*	*0.72*	*−0.065*	*1.59*
*Ch SurCBSD*	*Ch CBSD Max*^e^	*12.78*	*0.001*	*0.27*	*0.26*	*0.00040*
*Mk SurCBSD*	*Mk CBSD Max*	*45.13*	*<0.001*	*0.65*	*0.22*	*0.0016*
*All SurCBSD*	*All CBSD Max*	*22.16*	*<0.001*	*0.27*	*0.27*	*0.00045*
*Ch Sur 1*	*Ch CBSD Max*	*6.38*	*0.016*	*0.15*	*−0.010*	*4.42*
*Mk Sur 1*	*Mk CBSD Max*	*55.85*	*<0.001*	*0.70*	*0.19*	*1.39*
*All Sur 1*	*All CBSD Max*	*21.55*	*<0.001*	*0.26*	*0.20*	*1.49*

a Chato Surrounding CBSD.

b Mkuranga Surrounding CBSD.

c Both Chato and Mkuranga Surrounding CBSD.

d Chato Surrounding CBSD index 1.

e Maximum CBSD incidence (8MAP for Mkuranga and 7MAP for Chato).

## 4 Discussion

**Fig. 15:**
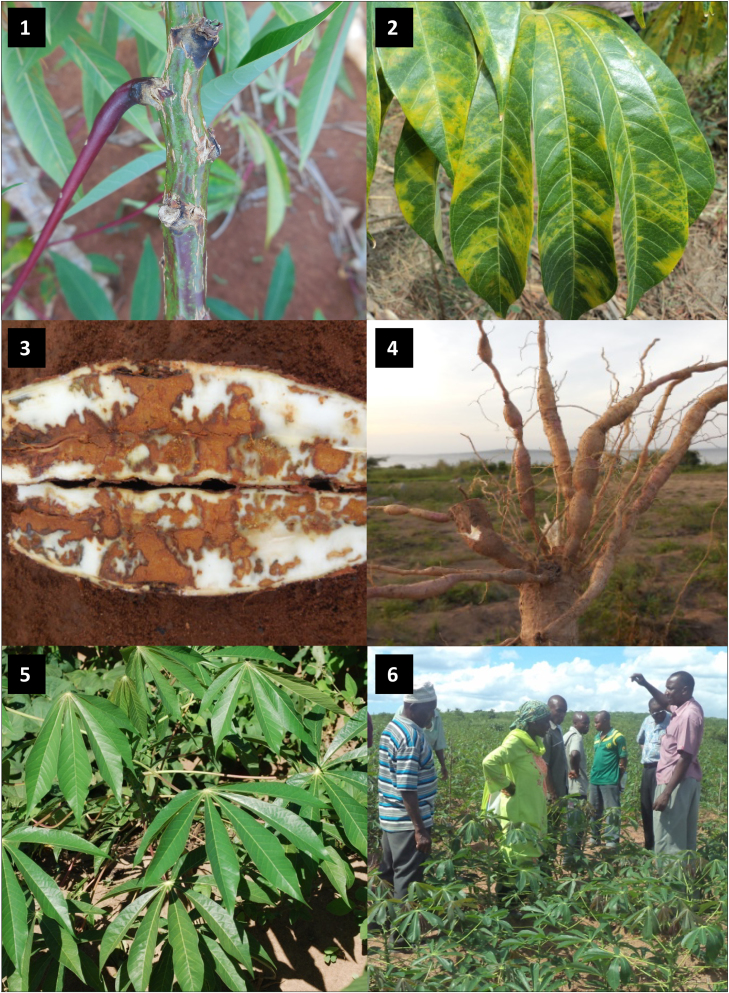
Community phytosanitation in Tanzania. 1. Necrotic lesions on cassava stem caused by CBSD; 2. Foliar symptoms of CBSD comprising a blotchy yellow chlorosis, particularly associated with secondary and tertiary veins, and most prominent on lower leaves of infected cassava plants; 3. Dry brown, corky necrosis in tuberous roots associated with severe CBSD infection; 4. Constrictions in tuberous roots which are a common feature of CBSD; 5. Healthy plants of the disease-free introduced variety − *Mkombozi*; 6. Community phytosanitation taskforce members explaining the objectives of the community phytosanitation initiative to cassava farmers in Chato, north-western Tanzania.

Cassava brown streak disease is an increasing threat to cassava production in East and Central Africa and has consequently been the subject of renewed research interest, particularly since the emergence of new epidemics in the Great Lakes region (Alicai et al., 2007, Ntawuruhunga and Legg, 2007, Legg et al., 2011). Much of the focus has been on methods to control the disease, and most of the research has pursued either conventional (Jennings, 1957, Kulembeka et al., 2012), or transgenic (Ogwok et al., 2012, Odipio et al., 2014) approaches to developing resistance. Data on the epidemiology of CBSD, however, has highlighted the fact that the disease spreads in the field over relatively short distances, and that CBSIs are transmitted by *B. tabaci* with a semi-persistent mechanism (Maruthi et al., 2005, Jeremiah, 2012). These biological characteristics highlight the potential value of phytosanitary measures as an effective component of disease management. The first epidemics of CMD in Uganda were successfully controlled through the large-scale implementation of phytosanitation that involved the complete removal of existing stocks of local susceptible cassava varieties and their replacement with disease-free planting material of an introduced CMD-resistant variety (Jameson, 1964). Based on its epidemiological characteristics, CBSD should be more readily managed with phytosanitation than CMD. This set of conditions provided the incentive for the planning and implementation of the first community-wide phytosanitation programme to manage cassava viruses in Africa since the 1940s (Fig. 15).

Tanzania was the first location in which CBSD was reported in the 1930s (Storey, 1936). The disease remained largely confined to coastal areas (Nichols, 1950) up until 2006 when new outbreaks were reported from the Lake Zone in the north-western part of the country (Ntawuruhunga and Legg, 2007, Jeremiah and Legg, 2008, Mbanzibwa et al., 2009, Legg et al., 2011). Incidences in the Lake Zone have increased rapidly in recent years (Jeremiah et al., 2015, Ndyetabula et al., 2016), and areas to the south and east of Lake Victoria have been most severely affected. The two zones selected for the community phytosanitation study in Tanzania corresponded to the endemic area in the eastern coastal part of the country (Mkuranga) and the recent outbreak zone on the southern shores of Lake Victoria (Chato). Ndyetabula et al. (2016) recorded incidences of 49.5% for the Coastal Zone and 32.7% for the Lake Zone. However, maximum district-level incidences were 92% for Kibaha District in the Coast Zone and 98% for Musoma District in the Lake Zone. CBSD total plant incidences determined in the current study, using the same methodology as that used by Ndyetabula et al. (2016), were closely comparable with these high incidences reported previously. Locations selected for the community phytosanitation study were therefore typical of high CBSD incidence areas of Tanzania and strongly representative of the more severely affected parts of the country.

### 4.1 Community phytosanitation produces an area-wide reduction in CBSD incidence

Following the implementation of the community phytosanitation approach in Chato and Mkuranga, there was a large reduction in CBSD incidence from >90% to <3%. This was achieved in both locations through the introduction to portions of the target community (Primary Recipient Groups − PRG) of certified disease-free planting material of improved varieties. Over the course of the study, which lasted for three seasons in Chato and two in Mkuranga, CBSD incidences increased gradually, although the incidence at the end of the third season in Chato was only 27%. Cassava farmers in East Africa typically cultivate a diversity of varieties (Balyejusa Kizito et al., 2007), each of which may have different uses. It is therefore unsurprising that there was a modest level of local variety re-introductions to the areas under community phytosanitation management. This material was similarly diseased to the plants that farmers were cultivating prior to the start of the programme, and the resulting increased incidence in the environment was an important factor in the gradual increase of CBSD in community phytosanitation managed zones. This factor highlights the importance of providing alternative variety options when introducing new varieties to an area where cassava is an important food staple and livelihood source. Overall, however, it can be stated that community phytosanitation provided a sustained reduction in CBSD incidence in the two target locations.

### 4.2 Phytosanitary measures implemented collectively by communities reduced CBSD incidence compared with unmanaged controls

Comparisons were made between improved varieties introduced under community phytosanitation conditions (PRG, SRG and TRG groups) and the same varieties from an identical source introduced under comparable conditions in a neighbouring community but without the phytosanitation (CRG). Final incidences of CBSD in the community phytosanitation treatments were significantly less than those in the unmanaged controls. In Chato, maximum incidence of CBSD was 0.6% in the community phytosanitation area while it was more than 60% in the CRG control, and in Mkuranga the reduction through community action was 81% compared to the control. These results highlight the effectiveness of the community management approach in reducing levels of disease over a single cropping season. The power of the approach is demonstrated further by the comparison between the maximum incidence in the third season of the PRG community phytosanitation group in Chato (36.9%) when compared with the >60% incidence in the unmanaged CRG group after a single season. In practical terms, this means that farmers could cultivate a relatively CBSD-susceptible variety such as *Mkombozi* for at least three seasons using the community phytosanitation approach and still have planting material that is significantly healthier than material that would be available to farmers cultivating the same variety for a single season but without community-based management. There are no data for community phytosanitation of cassava from any other source, so we are unable to confirm the efficiency of the approach with other instances elsewhere. However, work on other plant-disease-vector systems have demonstrated similar results. A globally important example is Huanglongbing (HLB) disease of citrus, which is spread by psyllid insect vectors, and causes hundreds of millions of US$ dollars’ worth of losses annually to the citrus industry. Detailed studies in Brazil compared local versus regional approaches to disease/vector management, and noted that regional management delayed epidemics by 10 months compared with local management controls and reduced disease incidence by up to 90% (Bassanezi et al., 2013). The authors concluded that area-wide inoculum reduction and psyllid management strongly affected HLB control and recommended the more widespread application of these approaches.

### 4.3 Community phytosanitation of CBSD can boost cassava yields

Substantial increases in yield were achieved by the farmer groups applying community phytosanitation with the introduced disease-free varieties of *Mkombozi* in Chato and *Kiroba* in Mkuranga. Overall yield increases ranged from a doubling to a greater than four-fold increase. This result is not altogether surprising, since both varieties were officially released by the Tanzania national research system on the basis of their high yielding potential. The more important comparison for this study was the yield produced by these improved varieties under community phytosanitation management versus the yield of the same varieties in neighbouring locations where phytosanitation was not practised. Under these circumstances, a significant yield increase (86%) was recorded for *Mkombozi* in Chato where community phytosanitation was applied, however, there was no significant difference between yields of *Kiroba* in Mkuranga under community phytosanitation managed and unmanaged conditions. In both locations, significant reductions in CBSD incidence were achieved through community phytosanitation. The failure of this to translate into a yield advantage for *Kiroba* in Mkuranga is almost certainly a consequence of its relative tolerance to CBSD (Maruthi et al., 2014, Kaweesi et al., 2014), in contrast to *Mkombozi* which is moderately susceptible. This has important implications, since this provides evidence suggesting that community phytosanitation can provide both reductions in CBSD incidence as well as yield increases where moderately susceptible varieties are introduced, but may only provide disease incidence reduction where the variety is tolerant to CBSD.

### 4.4 Community phytosanitation gives an area-wide reduction in CBSD inoculum pressure

Previously, manual approaches have been used to calculate inoculum pressure for cassava viruses, and these considered the number of infected plants within a radius of 250 m surrounding the target field, as well as the distance between surrounding fields and the target (Legg et al., 1997, Aritua et al., 1999). In the current study, we used two methods to determine inoculum pressure. The first was an electronic version of the Legg et al. (1997) method, in which physical measurement of distances between fields was substituted by estimates of distance calculated using GIS software, and based on GPS-derived coordinates for all cassava fields in the areas of study. The second method used CBSD incidence and vector abundance data to generate inoculum pressure index (Sur 1) values for each of the fields surrounding monitored plots. Kriging analysis was then applied to these point values to generate an interpolated geospatial surface which both demonstrated the spatial variation in inoculum pressure, as well as providing inoculum pressure index values for each of the monitored field locations. An important proviso that needs to be considered in utilizing these results is that caution should be exercised in interpreting the interpolated maps as samples were strongly clustered and large areas of the maps were not sampled. For areas within and immediately surrounding sampled points, kriging provides an accurate estimate of data values in intervening areas, however, predictions are less accurate in parts of the mapped area in which there are no data points, particularly towards the peripheral parts of the maps. In these areas predictions are largely extrapolations with limited accuracy. Nevertheless, several key statistics applied to these datasets highlight the significance in the reduction of CBSD inoculum pressure produced through the community phytosanitation approach. Firstly, the two inoculum pressure assessment techniques were strongly correlated, indicating that they are comparable measures. Secondly, both estimates of inoculum pressure strongly predicted the pattern of CBSD infection in the plots of farmers who cultivated initially healthy plantings of cassava − variety *Mkombozi* in Chato and *Kiroba* in Mkuranga. There has been limited previous study of the epidemiology of the CBSIs that cause CBSD, although the reports that are available suggest that the disease spreads over relatively short distances (10 s of metres) from nearby infected fields (Jeremiah, 2012). There are published data suggesting that *M. glaziovii* (Ceara rubber) is an alternative host for CBSIs, as well as other unpublished information indicating that several other plant species native to Tanzania may be infected by CBSIs. However, there is no indication so far that any of these plants are a significant source of CBSIs in the cassava farming environments of Tanzania, or anywhere else in Africa. Importantly, our data illustrate very clearly that CBSD inoculum in surrounding cassava fields is the key driver of CBSD spread into initially disease-free cassava plantings. This evidence reinforces the potential value of phytosanitation as an effective tool for the management of CBSD.

### 4.5 Potential for sustainability and scaling out of community phytosanitation

The pilot study of community phytosanitation to manage CBSD that is reported here was undertaken in two communities in different parts of Tanzania. This was achieved with support from a grant from an external funding source. In order for the approach to be of broader country-wide value, mechanisms would need to be identified that would enable the scaling of the approach through parts of the country where conditions are appropriate. This study demonstrated that community phytosanitation can deliver reduced inoculum levels of CBSD, reduced disease spread into initially CBSD-free plantings of new varieties and greatly increased yields for growers. However, important pre-conditions for success are likely to be: the severity of the CBSD disease problem, the importance of cassava in the livelihoods of the target community, the moderate susceptibility to CBSD of the introduced variety and the availability of sources of disease-free planting material. Community phytosanitation is unlikely to be sustained in the long-term in target areas, or scaled out to new target areas, in regions of coastal Tanzania where there is easy access to sources of planting material of the variety *Kiroba*, since this variety has a high level of tolerance to CBSD infection. The consequence of this is that farmers do not gain appreciably from planting CBSD-free cuttings of this variety, since infected plants are tolerant to CBSD and rarely express symptoms in the tuberous roots. As we have shown through this study, there is therefore little difference in the yield of CBSD-free versus CBSD-infected plants. By contrast, the prospects for sustained application of community phytosanitation and its scaling are much greater the in Lake Zone of Tanzania, where the improved variety that is most widely available is moderately susceptible to CBSD. It might be envisaged, therefore, the future programmes to manage CBSD could apply a twin-pronged approach in which efforts to breed for increasing levels of resistance/tolerance to CBSD are combined with programmes to promote community phytosanitation where such varieties are currently unavailable. In the longer term, however, it would be beneficial to incorporate both approaches, together with systems for the production of certified virus-tested planting material, into a holistic management approach that aims to minimize the impacts of cassava viruses. Even where varieties are tolerant to the effects of cassava viruses, propagating those varieties ensures that pathogenic viruses are sustained in the environment and have a greater likelihood of producing variants that might have even more damaging effects. An integrated management approach, that combines diverse approaches to virus and vector management, should be the long-term goal of countries and territories affected by cassava viruses.

## 5 Conclusion

A pilot study was undertaken at two locations in CBSD-affected parts of eastern and north-western Tanzania to investigate the potential value of community phytosanitation as an approach to manage the disease. The approach was shown to be effective in reducing CBSD inoculum pressure, sustaining low incidences of CBSD in newly-introduced planting material of improved varieties and in providing farmers with two to four-fold yield increases compared with previously cultivated local varieties. Newly introduced varieties performed significantly better under community phytosanitation conditions than they did when introduced to control farming environments where this approach was not practised. However, this benefit was only realized in one of the two communities, where the introduced variety was moderately susceptible to the disease. Community phytosanitation has been applied in other parts of the world for vector-borne plant pathogens, although it has been most commonly referred to as ‘area-wide disease management’. Notable successful examples are for the control of Laurel Wilt (*Raffaelea lauricola*) affecting avocadoes in Florida in the United States (Evans et al., 2015), rice tungro in south-east Asia (Cabunagan et al., 2001) and HLB in Brazil (Bassanezi et al., 2013). Moreover, it has been argued that there is a ‘critical importance of collective action’ for the management of many vector-borne diseases such as HLB or tomato-infecting begomoviruses, based on both practical and theoretical modelling considerations (Filho et al., 2016). All of the successful programmes for area-wide disease management have focused on highly commercialized crops being cultivated by well-informed networks of growers. The challenge for the wider application of community phytosanitation for the management of CBSD in Africa, will be in identifying the appropriate scaling conditions for this strategy that has been shown through the current study to be highly effective in reducing the local impacts of the disease.

## Conflict of interest

The authors declare no conflicts of interest.

## Funding

This work was funded through a grant from the Bill and Melinda Gates Foundation to Tanzania’s Department for Research and Development of the Ministry of Agriculture, Livestock and Fisheries. The participation of IITA scientists was additionally supported by the CGIAR Research Programme on Roots, Tubers and Bananas.
